# Case of Dropsy of the Pericardium

**Published:** 1829-05

**Authors:** F. W. Wood


### DROPSY OF THE PERICARDIUM.

*Case of Dropsy of the Pericardium.* By F. W. Wood, Esq.

William Fornsett, aged twenty-six, by employment a husbandman, applied for medical aid, 19th December, 1828. He is of a sanguineo-bilious temperament. Pulse ninety-eight, hard, sharp, and peculiarly vibrating; tongue furred and brown; mouth clammy, with a coppery taste; bowels constipated; great tenderness over the epigastric region, and along the margin of the liver; urine high coloured, with a deep red sediment; pain in the head, shoulders, and legs; difficult respiration, and constant dry cough; no appetite. Has been ill nearly a year. His family report that he has been an habitual drunkard for many years.

*Treatment.*—V.S. ℥xviij. Blood buffed and cupped, serum yellow green. ℞. Hyd. Submur. gr. vj.; Ext. Coloc. comp. gr. xij. M. fiat pil. iij. stat. sumend.—℞. Hydrarg. Submur. gr. iss.; Aloes purif. Ext. gr. iss. ; Sapo dur. q. s. fiat pil. omni nocte sumend.

20th.—Slight amendment. From this time until January 19th, he fell into other hands, and was principally treated for some presumed pulmonic affection; when, finding himself much worse, he returned to my care.

Jan. 19.—Pulse 130, full, hard, and vibratory, resembling the blow of a liberated spring against the finger, and distinctly *audible* at the forearm and along the thigh. Upon using the stethoscope, the noise in the heart resembled the escape of steam from a tube. Pulsation of the carotid and temporal arteries visible. Head free from pain; bowels constipated; urine scanty and high coloured; abdomen swelled and tense, accompanied with tenderness; cough frequent and harsh, expectorating a thin glairy fluid in small quantities.

V.S. ℥xxxij. Blood sizy, serum tinged green, much cupped; appearance, while flowing, dark and muddy.—℞. Aloes purif., Hydrarg. Submur. *aa* gr. iij.; Pulv. G. Resin Scamm., Ext. Coloc. c. *aa* gr. iv. M. fiant pil. ij. s. s. —℞. Pil. Hydr. gr. iv. ter in die sumend. cum Pulv. Digitalis gr. $\frac{1}{2}$; Pulv. Tragacantha c. gr. v.—Milk diet, and to be kept still.

20th.—Not seen.

21st.—Pulse eighty; breathing relieved; pain in the epigastric region much diminished; evacuations plentiful, but dark and fetid; cough less frequent; sleeps tolerably well; countenance sallow, but without that expression of anxiety so strongly depicted on the 19th.—Medicines cont.

23d.—No pain; pulse increasing, 98 to 100; evacuations dark coloured and scanty.

24th and 25th.—No perceptible alteration.

26th.—Mercurial action taken place; slight ptyalism; bowels obstinately constipated; pulse 100, hard and vibratory.—℞. Ext. Coloc. c. gr. xv. fiant pil. iij. s.s.—℞. Magn. Sulph. ʒi.; Pulv. Digitalis gr. $\frac{1}{2}$, fiat pulv. ter in die sum.

27th, 28th, 29th.—Reported much the same.

Jan. 30th.—Mercurial action subsiding; pulse 100; respiration difficult; bowels inactive; tongue furred; restlessness, cough, and thirst.—℞. Magn. Sulph. ℥ij.; Mist. Camph. ℥iv.; Infus Sennæ ℥ij.; Tr. Digitalis ʒiss. M. cap. ¼ partem quarta quaque hora donec, mag. denide cap. coch. responderit alvus quartis horis.—V.S. ℥xij

31st.—To take the mixture, (coch. mag. ij. tertiis horis.) Bowels freely relieved: stools dark and fetid, improving after the early dejections; copious sediment in the urine; pulse ninety-four, character as before.

February 1st to 4th.—Ordered the mixture occasionally. Pulv. Digitalis increased to three grains three times a day.

5th.—Pulse 90, volume slightly diminished; bowels regular; dejections healthy; pain throughout the thoracic cavity; countenance tinged yellow; eyes languid, but bright; appetite (for the first time) moderate; evident prostration of strength. Seen at intervals, until placed under Dr. Yates' care. Ordered, Pulv. Potas. Supertart. ʒvj. solve in aquæ ℔iss. cap. ℥ij. ter in die; adde Tr. Digitalis ♏x. singulis dosibus —℞. Ext. Hyoscyami, Pil. Hydrarg. *aa* gr. xij. Misce et div. in pil. equales vj. sumat i. omni nocte. —Empl. Lyttæ sterno.

Dr. Y. expressed it as his opinion that there was organic disease of the heart.

The patient continued the use of these means for three weeks. He was then confined to his bed. Pulse now 100 and upwards, character the same, but less energy; legs œdematous; face pale, lips lead coloured; cough violent; sleeps in a perpendicular posture; sense of general uneasiness; very restless. Relieved by small doses of compound spirit of æther. He lingered for a few days, when death terminated his sufferings.

Permission having been obtained to examine the body, it was opened on the fourth morning after his decease. Upon turning back the sternum, the attention of myself, Dr. Stone, and Mr. Lucas, was instantly attracted by the appearance of a large bladder, occupying the whole of the left and nearly two parts of the right side of the thoracic cavity. Further investigation discovered this to be the pericardium, which was distended to this extraordinary size. Upon carefully emptying its contents, it was found to contain two quarts of a perfectly limpid fluid. The outer coat of the pericardium had a thin shining appearance, whilst on the inside was deposited layers of coagulable lymph, resembling the rugæ on the stomach of a cow. The heart was also covered with a similar deposit; its parietes were much thickened, and it appeared as if foreshortened, the apex being pressed upwards. In the left ventricle was a portion of coagulable lymph, of a yellow sizy appearance, of nearly an ounce weight. The carneæ columnæ were much enlarged; the valves free from any apparent disease. The structure of the lungs was perfectly unimpaired.

Abdominal cavity: Stomach and intestines quite healthy; liver rather enlarged, and somewhat indurated; gall bladder filled with healthy bile; kidneys and spleen healthy.

The head was not examined, as there were no indications of disease in the brain.

It may be worthy of observation, that the mother of this patient died of ascites.

*Tunbridge Wells, Kent.*

The quantity of fluid effused between the pericardium and the heart in this case is very remarkable. Dr. BAILLIE observes, in speaking of dropsy of the pericardium, ("Morbid Anatomy," Wardrop's edit. vol. ii. p. 8,) "This water varies a good deal in quantity, amounting in some cases hardly to two ounces, and in others to more than a pint. Although the quantity accumulated be large, yet the pericardium is never very much stretched, but always appears as if it could contain a greater quantity. It is probable, therefore, that the pericardium may really grow so as to keep pace with the accumulation; and this would seem to be necessary, in order that the heart may have room for dilating its several cavities."—EDITORS.

---

